# Time-Dependent Effects of Localized Inflammation on Peripheral Clock Gene Expression in Rats

**DOI:** 10.1371/journal.pone.0059808

**Published:** 2013-03-20

**Authors:** Susan Westfall, Argel Aguilar-Valles, Valérie Mongrain, Giamal N. Luheshi, Nicolas Cermakian

**Affiliations:** 1 Douglas Mental Health University Institute, Montreal, QC, Canada; 2 Department of Neurology and Neurosurgery, McGill University, Montreal, QC, Canada; 3 Department of Psychiatry, McGill University, Montreal, QC, Canada; Vanderbilt University, United States of America

## Abstract

Many aspects of the immune system, including circulating cytokine levels as well as counts and function of various immune cell types, present circadian rhythms. Notably, the mortality rate of animals subjected to high doses of lipopolysaccharide is dependent on the time of treatment. In addition, the severity of symptoms of various inflammatory conditions follows a daily rhythmic pattern. The mechanisms behind the crosstalk between the circadian and immune systems remain elusive. Here we demonstrate that localized inflammation induced by turpentine oil (TURP) causes a time-dependent induction of interleukin (IL)-6 and has time-, gene- and tissue-specific effects on clock gene expression. More precisely, TURP blunts the peak of *Per1* and *Per2* expression in the liver while in other tissues, the expression nadir is elevated. In contrast, *Rev-erbα* expression remains relatively unaffected by TURP treatment. Co-treatment with the anti-inflammatory agent IL-1 receptor antagonist (IL-1Ra) did not alter the response of *Per2* to TURP treatment in liver, despite the reduced induction of fever and IL-6 serum levels. This indicates that the TURP-mediated changes of *Per2* in the liver might be due to factors other than systemic IL-6 and fever. Accordingly, IL-6 treatment had no effect on clock gene expression in HepG2 liver carcinoma cells. Altogether, we show that localized inflammation causes significant time-dependent changes in peripheral circadian clock gene expression, via a mechanism likely involving mediators independent from IL-6 and fever.

## Introduction

Circadian clocks allow living organisms to adapt their internal biology to the 24 h day. Variations in environmental cues (or Zeitgebers) such as the light-dark cycle (LD), feeding times or socialization rhythms coordinate molecular circadian oscillations to daily cycles. Circadian clocks are endogenous to most cells in the body and their coordination is essential for internal homeostasis and health [1]. The master pacemaker resides in the suprachiasmatic nucleus (SCN) of the anterior hypothalamus and is necessary for the synchronisation of circadian oscillators located in other brain regions and most peripheral tissues [1]. The central SCN clock elicits coordination of peripheral clocks through direct neuronal mechanisms [2], [3] and humoral mechanisms [4]–[6]. The molecular clock is composed of clock genes including *Clock, Bmal1, Cryptochromes (Cry)1* and *2*, *Periods (Per)1,* and *2* and the orphan nuclear receptors *Rev-erbα* and *Retinoid acid-related orphan receptor (ROR) α*. These clock genes are involved in self-sustaining transcriptional feedback loops that occur with a period of approximately 24 h [7].

As is the case for various other regulatory systems, many aspects of the immune system show 24 h rhythms, including circulating cytokine levels and counts of various immune cell types [8], [9]. In general, proinflammatory factors peak during the rest phase, which is the dark period in humans and the light period in nocturnal rodents [10]. Circadian rhythmicity of symptoms is common in inflammatory diseases [11]. For example, patients suffering from sepsis have the highest susceptibility to mortality in the early morning [12], while patients suffering from rheumatoid arthritis, bronchial asthma or ankylosing spondylitis experience their worse symptoms early in the morning [13]–[15]. In rodent models, treatment with the bacterial cell wall component lipopolysaccharide (LPS) during the rest period (light phase) invokes a substantially higher mortality risk compared to animals treated in the active period (dark phase), paralleling peak cytokine induction [16], [17].

In addition to this circadian control of inflammatory processes, inflammatory stimuli can impose changes in both central and peripheral circadian clocks. In humans, LPS administration suppresses clock gene expression in peripheral blood leukocytes [18] Similarly, rodents treated with LPS show suppressed molecular rhythms in both central and peripheral clocks [19], [20] and a phase shift of behavioural circadian rhythms [21].

Different inflammatory mediators were proposed to influence central and peripheral clocks [22]–[24], which could be responsible for the circadian disruptions following an endotoxin challenge. For instance, IL-6 was shown to stimulate *Per1* promoter activity in cultured cells [25], but nothing is known on its effect on clock genes *in vivo*. Tumor necrosis factor alpha (TNFα) time-dependently suppresses the expression of many clock genes both in cultured cells and *in vivo* [22] and suppresses melatonin in the pineal gland [26]. Finally, interferon alpha (IFNα) suppresses *Per1* in mouse liver and adrenal glands, but only with treatment during the active phase [24].

In this study, we address the effects of localized inflammation induced with turpentine oil (TURP) treatment on peripheral clock gene expression. An intramuscular TURP injection creates a sterile abscess and inflammation characterized by local synthesis of IL-1β and TNFα [27], [28]. These cytokines do not enter circulation, but induce the synthesis of IL-6, which then induces cyclooxygenase (COX)-2 gene transcription and prostaglandin (PG)E_2_ production in the brain thermoregulatory centres, leading to fever development [29]. Compared to LPS, TURP presents clear advantages to decipher the mechanism behind the effects of inflammation on circadian clocks. First, TURP elicits a simpler cytokine response, with IL-6 being the only circulating proinflammatory cytokine. Second, the slower time-course of TURP (6 h–10 h to reach maximal IL-6 levels and fever) compared to LPS (2 h–4 h) allows correlations to be made between inflammatory mediators and changes in clock gene expression. Using this strategy, we show that TURP elicits time-, tissue- and gene-specific effects on peripheral clock gene expression. Further, we investigated the possible mechanisms underlying these effects both *in vivo* and *in vitro* and found that despite the correlative peak changes of IL-6 with *Per* gene expression in the liver and heart, IL-6 does not seem to act directly to mediate these effects.

## Materials and Methods

### Ethics Statement

All procedures with animals were approved by Douglas Institute Facility Animal Care Committee and McGill University Animal Care Committee, in line with the guidelines of the Canadian Council on Animal Care.

### Animals

Adult male Sprague-Dawley rats (2–3 months old, weighing 250–300 g at the time of experiment; Charles River, Saint Constant, QC, Canada) were used in the experiments. The animals were housed individually in a controlled environment with a regular 12 h:12 h LD cycle (∼200 lux in the light periods) in an ambient temperature of 21±2°C. Regular chow and water were available *ad libitum*.

### Treatments

In all experiments, animals were treated with either 0.1 mL of purified turpentine oil (TURP) (Riedel-deHaen, Sleeze, Germany) or 0.1 mL of sterile physiological saline by intramuscular (I.M.) injection into the right gastrocnemius muscle (hind-limb).

In one experiment, animals were treated with human recombinant (hr) IL-1Ra (provided by Dr. Stephen Poole, National Institute for Biological Standards and Control, Hertfordshire, UK). Animals were treated intraperitoneally (I.P.) at a dose of 1 mg/kg at 0, 4, 8, and 12 h after TURP treatment. The hrIL-1Ra was dissolved in physiological saline to a concentration of 1 mg/mL, and control animals were treated similarly with an equal volume of physiological saline.

The times of treatment and sacrifice varied depending on the experiment. Animals were sacrificed by decapitation, blood was collected by trunk bleed into sterile tubes and serum was isolated by immediate centrifugation at 4000 rpm and 4°C for 10 min. Aliquots were made and samples frozen at −80°C until use. Livers, hearts, kidneys and spleens were collected upon sacrifice, frozen on dry ice and stored at −80°C until use.

Analgesics were not used in our study, primarily because of the potential impact on some of our endpoint measures: Analgesics were shown to alter inflammatory responses and to affect the levels of pro-inflammatory cytokines [30], [31]. Moreover, analgesic treatment has impacts on circadian rhythms and on clock gene expression [32], [33]. Additionally, the pain experienced by the rats upon injection of the dose of TURP used in our study (0.1 mL) is relatively minor, compared to the effects of the higher dose of 0.6 mL of TURP used in some of our previous studies [34], and the animals did not exhibit any outward signs of distress nor any pain-induced vocalization.

All injections during the dark phase (ZT12-ZT0) were performed under dim red light. When animals were to be sacrificed during the dark phase, they were taken out of the room in a lightproof box and were not exposed to light until less than a minute before sacrifice.

### Temperature Recordings

Temperature was monitored using remote radio-biotelemetry (Data Science International, St Paul, MN, USA) as previously described [35]. Briefly, two weeks before the experiment, animals were abdominally implanted with pre-calibrated temperature-sensitive radio transmitters (TA10TA-F40, Data Sciences International) under isoflurane anaesthesia. Two days before the experiment, animals were placed on individual receiver boards containing an antenna to monitor the temperature output frequency (Hz) every 10 min until sacrifice. The temperature frequency was converted into degrees centigrade with Dataquest software (Data Sciences International). The baseline 24 h temperature fluctuation was established two days prior to treatment in each animal to ensure a normal diurnal profile.

### *In vivo* Experiments

Before each of the following three experiments, animals were habituated for 2 weeks in a 12 h:12 h LD cycle with daily handling at least one week before the experiment. This involved taking the animals out of their cages and manipulating them in the same way they would be at the time of injection (i.e., for handling prior to I.M. injection, animals held at the side of the experimenter and their rear gastrocnemious muscle revealed; prior to I.P. injections, animals wrapped in the same towel as for the injections and positioned such as to receive an injection).

#### Experiment 1: The effect of a morning TURP injection on clock gene expression

Animals were treated with either TURP or saline at ZT2 (2 h after lights on given that Zeitgeber Time 0 [ZT0] corresponds to lights on and ZT12 to lights off). Animals were then sacrificed 2, 6, 10, 14, 18, and 22 h after treatment (i.e. ZT4, ZT8, ZT12, ZT16, ZT20 and ZT0 time of sacrifice (TOS), respectively). Each group included 4 animals. All measurements for clock genes and cytokines were performed at the TOS.

#### Experiment 2: The effect of injection time on TURP-induced changes of clock gene expression

Animals were treated with either TURP or saline at 4 time points over 24 h: ZT2, ZT8, ZT14 and ZT20 time of injection (TOI). Animals were then sacrificed exactly 10 h after the TOI (i.e., ZT12, ZT18, ZT0 and ZT6 TOS, respectively) as this time corresponds with the maximal fever response in response to TURP [27]. There were 5 animals per time point each for TURP and saline. Measurements for clock genes and cytokines were performed at the TOS.

#### Experiment 3: The effects of anti-inflammatory IL-1Ra treatment on TURP-induced changes of clock gene expression

Animals were treated with either TURP or saline at ZT2 and sacrificed at ZT12 or ZT16 (10 h and 14 h after TOI, respectively). In each of the injection groups, animals were treated with either hrIL-1Ra or saline making a total of 4 groups per time of sacrifice (saline-saline, saline-hrIL-1Ra, TURP-saline, and TURP-IL-1Ra). The ZT12 group had a total of 3 hrIL-1Ra treatments at 0, 4 and 8 h after TOI (i.e., ZT2, ZT6, and ZT10, respectively) and the ZT16 group had 4 hrIL-1Ra treatments at 0, 4, 8 and 12 h after TOI (i.e., ZT2, ZT6, ZT10 and ZT14, respectively). Given the short half-life of IL-1Ra, multiple injections were required for a full effect on fever and IL-6 [36]. Further, IL-1Ra blocks the upregulation of IL-6 and to completely attenuate its response, IL-1Ra must be administered early in the TURP time-course when IL-6 begins to be produced [36].

### Cell Culture Experiment

The human carcinoma HepG2 cells were provided by Dr. Cindy Goodyer (McGill University, Montreal). Cells were seeded in 6-well plates at a density of 0.3×10^6^ cells per plate in Dulbecco’s modified Eagle’s medium (DMEM) (Life Technologies, Burlington, ON) supplemented with 10% fetal bovine serum (FBS) (Life Technologies), 2 mM L-glutamine (Life Technologies), and 100 mg/mL penicillin-streptomycin and grown to confluency at 37°C in 5% CO_2_. One day (24 h) before the first time point, growth media was replaced with a starvation media containing 0.5% FBS. On the day of the experiment, cells were transferred to a serum-free media containing the various concentrations of human recombinant IL-6 (CedarLane, Burlington, ON). Cells were then washed with cold PBS, lysed in Trizol (Life Technologies), and frozen at −80°C until use.

### Cytokine Assays

Serum concentrations of IL-6, IL-1Ra and TNFα were obtained in duplicate using an in-house-developed sandwich enzyme-linked immunosorbent assay (ELISA) (secondary enzymes supplied by NIBSC, Potters Bar, UK) as previously described [35]. The detection limit was between 30 and 50 pg/mL for IL-6, TNFα and IL-1Ra, as indicated in figure legends. The inter-assay variability was below 4% and the intra-assay variability was below 9% for all experiments.

### Clock Gene Expression

RNA was extracted using Trizol (Life Technologies) according to the manufacturer’s protocol. cDNA was synthesized using the MultiScribe reverse-transcription kit (Life Technologies) according to manufacturer’s instructions. In all rat tissues, clock gene expression was assessed using SYBR Green quantitative PCR (qPCR) (Life Technologies, 7500 Real-Time PCR System). Primers for clock genes *Per1, Per2* and *Rev-erbα*, were designed using Primer3 software (sequences in Table S1). The control primers tested were *Histone H1, HPRT (hypoxanthine-guanine phosphoribosyl transferase), GAPDH (glyceraldehyde-3-phosphate), Ubi (ubiquitin), and Tbp (TATA box binding protein)* (sequences in Table S1) [37]. In order to determine the most stable control genes over time and condition the GeNorm v3.3 software was used [38]. For each experiment conducted, two control genes were selected (Table S2).

In the human HepG2 cell line, mRNA expression was assessed with quantitative PCR using TaqMan probes (Life Technologies) as outlined by the manufacturer. From a set of 5 genes (*B2M; beta-2-microglobin, PPiA; peptidylprolyl isomerase A, TBP; TATA-binding protein, RPLPO; large ribosomal protein, GAPDH; glyceraldehyde-3-phosphate dehydrogenase, ACTB; beta-actin*), the most stable control genes over both time and treatment were determined using GeNorm as described above: *B2M* (H200984230_m1) and *PPiA* (Hs04194521_s1). For clock gene expression, the following probe sets were used: *Per1* (H200242988_m1), *Per2* (Hs00256143_m1), *Rev-erbα* (Hs00253876_m1), *Serum amyloid A2* (Hs00605928_g1) and *Haptoglobin* (Hs01667582_m1). The relative mRNA expression of each clock gene was assessed by normalizing to the geometric mean of the two control genes, and then to a reference sample, using the 2^−ΔΔCT^ method [39].

### Statistical Analysis

In Experiments 1 and 2, the temperature curve differences, cytokine induction over time and changes in clock gene RNA levels were analyzed by two-way ANOVAs with factors Time and Condition. In Experiment 3, the variation in temperature over time in all the groups was analyzed with a three-way ANOVA with factors Time and Condition for TURP and Condition for IL-1Ra; and group differences in cytokine induction and clock gene expression were analyzed with a one-way ANOVA. Finally, clock gene expression in HepG2 genes was analyzed using two-way ANOVAs with factors Time and Condition. For ANOVAs, significant effects were decomposed using Tukey’s post hoc analysis for pair-wise comparisons when applicable. Induction of *SAA2* and *HP* gene expression in HepG2 cells was analyzed with Student t-tests. Statistical significance was set to *p*<0.05 and results are reported as mean ± SEM.

## Results

### The Effect of TURP Treatment on Fever, Cytokine Induction and Peripheral Clock Gene Expression *in vivo*

In Experiment 1, the effect of TURP on the rhythmic expression of clock genes in four peripheral tissues (liver, heart, kidney, spleen) was examined. Animals were treated with either TURP or saline early in the light phase at ZT2 (2 h after lights on) and were sacrificed 2, 6, 10, 14, 18 and 22 h after treatment (ZT4, ZT8, ZT12, ZT16, ZT20 and ZT0, respectively).

In Experiment 2, the effect of TURP on fever, cytokine levels and peripheral clock gene expression was compared for different injection times. Animals were treated with TURP at ZT20, ZT2, ZT8 and ZT14 times of injection (TOI) and sacrificed 10 h later at ZT6, ZT12, ZT18 and ZT0 times of sacrifice (TOS), respectively.

#### TURP induces a characteristic rise in temperature

**Figure 1:**
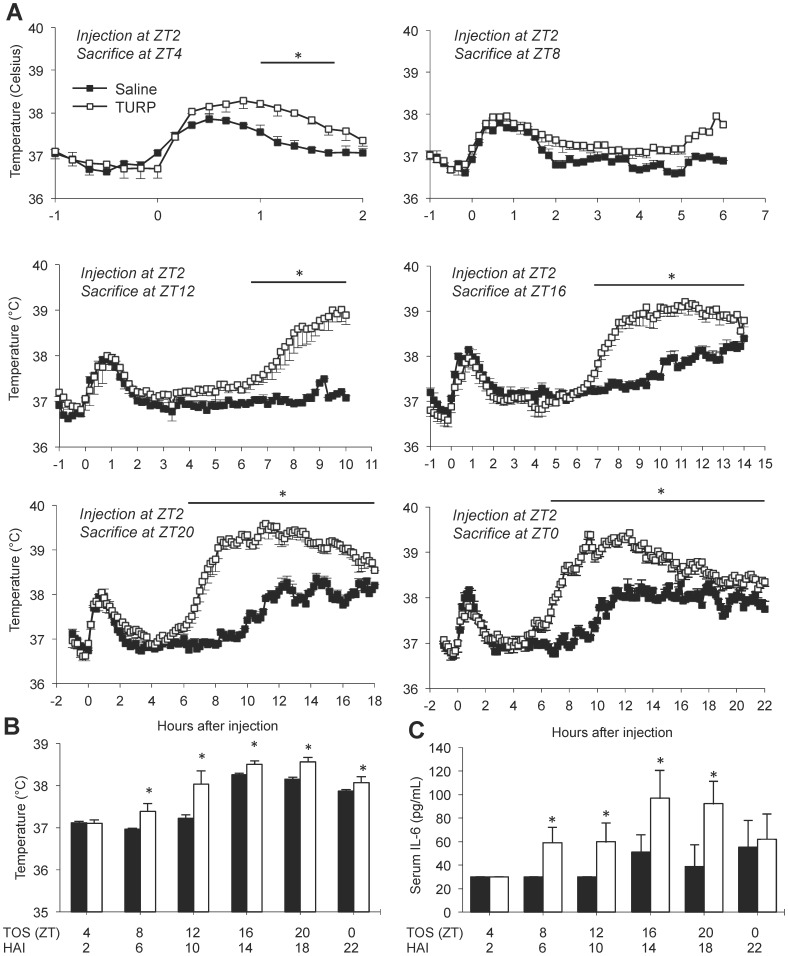
Fever and IL-6 induction over time after TURP treatment at ZT2. Animals were treated with either saline or TURP at ZT2 (0 hours) and sacrificed at the 6 different time points, ZT4, ZT8, ZT12, ZT16, ZT20, and ZT0 (time of sacrifice, TOS), which correspond to 2, 6, 10, 14, 18, and 22 h after injection (HAI), respectively. (A) Core body temperature was monitored beginning 1 h before treatment and every 10 min after treatment until sacrifice. Closed boxes represent saline-treated animals and open boxes represent TURP-treated animals. Two-way ANOVA Time×Condition *p*<0.001 at all TOS, except ZT4 (*p*<0.05) and ZT8 (*p*>0.05); Tukey post hoc tests between saline and TURP-treated groups (done for the graphs where there was a Time×Condition interaction) *p<0.05; n = 4. (B) The temperature at sacrifice was averaged between all animals in the last 30 min (3 time points). Closed boxes represent saline-treated animals and open boxes represent TURP-treated animals. Two-way ANOVA Time×Condition *p*<0.001; Tukey post hoc tests between the saline- and TURP-treated groups *p<0.05; n = 4. (C) Serum IL-6 levels at sacrifice were measured by ELISA. The assay detection limit is 30 pg/mL. Closed boxes represent saline-treated animals and open boxes represent TURP-treated animals. Between the saline- and TURP-treated groups *p<0.05; n = 4. Clock gene expression data for this experiment can be found in Fig. 3.

**Figure 2:**
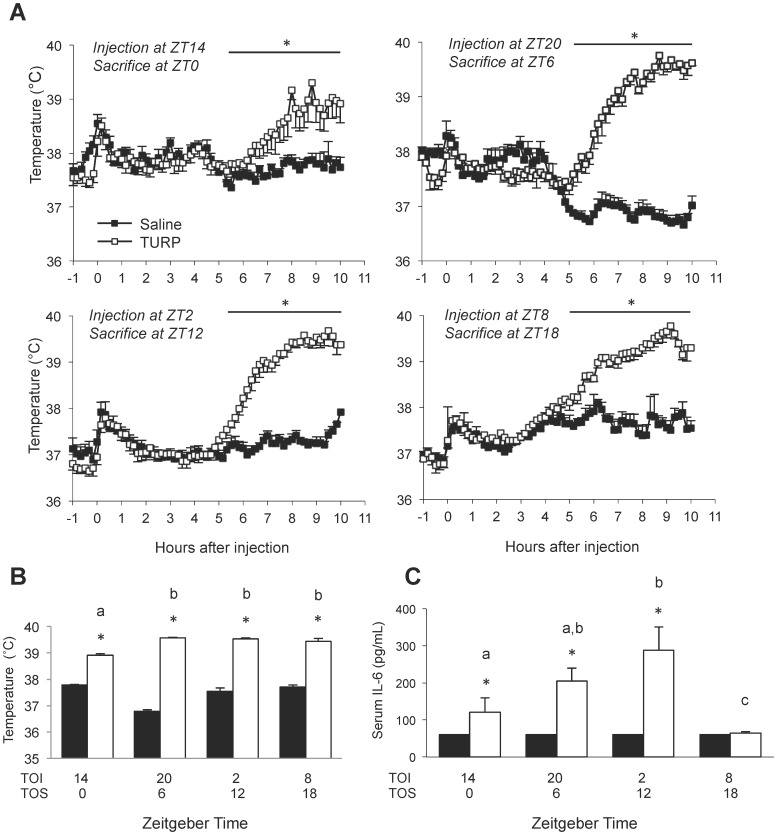
Time-dependent effect of TURP treatment on fever induction and IL-6 levels. Animals were treated with either TURP or saline at ZT14, ZT20, ZT2 or ZT18 (time of injection, TOI) and sacrificed 10 h later at ZT0, ZT6, ZT12 and ZT18 (time of sacrifice, TOS), respectively. (A) Core body temperature was monitored beginning 1 h before treatment and every 10 min after treatment until sacrifice. Animals were treated and sacrificed at the indicated time points. Closed boxes represent saline-treated animals and open boxes represent TURP-treated animals. Two-way ANOVA Time×Condition *p*<0.001 at all time points; Tukey post hoc tests between saline- and TURP-treated groups *p<0.05; n = 5. (B) The temperature at sacrifice of the last three recordings (30 min before sacrifice) was averaged among all animals in each group. Closed boxes represent saline-treated animals and open boxes represent TURP-treated animals. Two-way ANOVA Time×Condition *p*<0.001; Tukey post hoc tests between saline- and TURP-treated groups *p<0.01; between time points, different letters *p*<0.05, share one letter *p*>0.05; n = 5. (C) Serum IL-6 levels at sacrifice were measured by ELISA. The assay detection limit was 50 pg/mL. Closed boxes represent saline-treated animals and open boxes represent TURP-treated animals. Two-way ANOVA Time×Condition *p*<0.001; Tukey post hoc tests between the saline- and TURP-treated groups *p<0.05; between time points, only different letters *p*<0.05, share one letter *p*>0.01; n = 5. Clock gene expression data for this experiment can be found in Fig. 4.

In both Experiments 1 and 2, TURP treatment induced a significant increase of temperature compared to the saline controls (Figs. 1A, B, 2A, B). The temperature began to rise significantly 5–6 h after injection and reached a plateau at 8–9 h, as expected based on previous studies [27]. In Experiment 1, when the temperature in the last 30 minutes before sacrifice was considered, the magnitude of fever induction at sacrifice showed a significant Time×Condition interaction (*F*_5,115_ = 19.85, *p*<0.001) (Fig. 1B). The maximal difference between the saline- and TURP-treated groups occurred 10 h after injection (i.e. ZT12 TOS) (Fig. 1B). This rise began to subside 14 h after treatment and was back to nearly baseline levels after 22 h (Fig. 1A, B). This is consistent with previous studies [27].

Interestingly, in Experiment 2, while the magnitude of fever induction at sacrifice showed a significant Time×Condition interaction (*F*_3,99_ = 19.91, *p*<0.001) (Fig. 2B), post hoc analysis revealed that the temperature at sacrifice was mainly independent of the TOI, except for animals sacrificed at ZT0, where the temperature was slightly lower compared to all other TURP-treated groups (Fig. 2B).

The rapid and transient increase in temperature observed in all experiments right after injection of TURP or saline is due to a stress response consequent to both handling and injection. Since this increase is seen in both the saline and TURP groups and is of lower magnitude than the TURP-induced fever, and given the long time course of cytokines and fever in the TURP model (6–10 h before reaching a peak response), this initial transient increase in temperature did not likely affect our observations and conclusions.

#### TURP treatment leads to a time-dependent induction of IL-6

It was shown that serum IL-6 begins to rise 4 h and peaks 6 h after TURP treatment, slightly preceding the fever profile [27]. Other cytokines, namely IL-1β and TNFα are not released into the serum, but can be locally synthesized in a tissue-specific manner [40], [41]. In Experiment 1, IL-6 showed a rise in response to TURP starting at ZT8 TOS, 6 h after treatment (main effect of treatment, *F*_1,32_ = 7.48, *p*<0.05; Fig. 1C). Although the Time×Condition interaction was not significant (*F*_5,32_ = 1.05, *p*>0.05), there was a significant variation over time in the TURP group (*F*_5,32_ = 2.28, *p*<0.01), but not in the saline control (*F*_5,32_ = 0.649, *p*>0.05). Post hoc analysis revealed a significant TURP-induced increase in IL-6 over the saline controls 6, 10, 14 and 18 h after injection (ZT8, ZT12, ZT16 and ZT20 TOS, respectively; *p*<0.05), and was back to baseline levels after 22 h (ZT0 TOS). This profile parallels the increase in temperature.

In Experiment 2, there was a significant Time×Condition interaction of IL-6 levels at sacrifice when TURP was injected 10 h earlier (*F*_3,31_ = 5.76, *p*<0.001; Fig. 2C). At TOS ZT0, ZT6 and ZT12, IL-6 was significantly increased compared to saline controls (p<0.05), but not at ZT18. Also, the induced levels of IL-6 varied significantly over time in the TURP-treated group only (*F*_3,31_ = 11.53, *p*<0.001), with IL-6 levels in the TURP-treated group being significantly higher at TOS ZT12 than at TOS ZT0 or ZT18 (*p*<0.05).

Serum levels of IL-1Ra and TNFα were also measured at sacrifice. Previous reports have shown that upon TURP treatment, IL-1Ra is induced while TNFα is unaltered [27], [41]. Accordingly, comparable to IL-6, IL-1Ra rose following TURP injection when the TOS was in the early active phase (ZT12-18) in both Experiments 1 and 2 (Figs. S1A and S2A), but a significant Time×Condition interaction was found only in Experiment 2. TNFα levels were significantly elevated above saline controls in Experiment 1 at ZT4, ZT12 and ZT0 (2, 10 and 22h after injection, respectively) (Fig. S1B, *p*<0.05) and in Experiment 2 at ZT0 TOS (Fig. S2B, *p*<0.05). This induction, although significant, is several times smaller than TNFα induction usually observed in inflammatory models [42].

#### In vivo experiment 1: The effect of a morning TURP injection on clock gene expression

**Figure 3:**
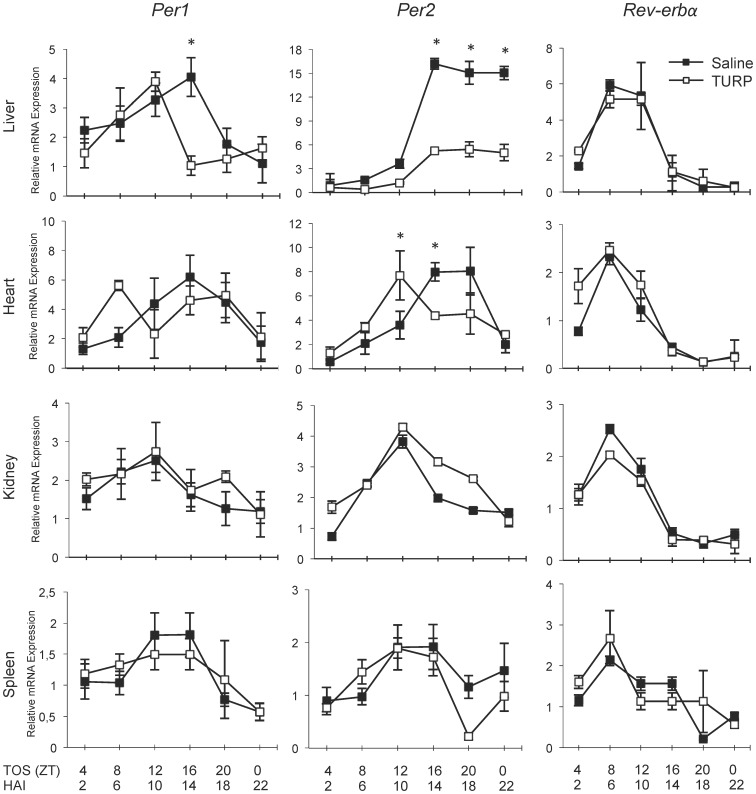
Clock gene expression over time after TURP treatment at ZT2. Animals were treated with either saline or TURP at ZT2 (0 h) and sacrificed at the 6 different time points, ZT4, ZT8, ZT12, ZT16, ZT20, and ZT0 (time of sacrifice, TOS), which correspond to 2, 6, 10, 14, 18, and 22 h after injection (HAI), respectively. Tissues were harvested upon sacrifice and RNA extracted. The relative expression of the clock genes *Per1, Per2* and *Rev-erbα* in the liver, heart, kidney and spleen is represented. Gene expression is relative to one sample in the ZT2 sacrifice group. Closed boxes represent saline-treated animals and open boxes represent TURP-treated animals. Two-way ANOVA Time×Condition *p*<0.05 for *Per1* in liver and heart and *Per2* in liver, *p*>0.05 for all others (p = 0.0532 for *Per1* in the heart); Tukey post hoc tests between saline and TURP-treated groups at each time point (done for graphs where there was a Time×Condition interaction) *p<0.05; n = 4. Body temperature and cytokine data for this experiment can be found in Figs 1 and S1.

Time-, tissue-, and gene-specific changes in clock gene expression were noted following TURP treatment in the early light phase (ZT2) (Fig. 3). In the liver, there was a significant Time×Condition interaction for *Per1* (*F*_3,31_ = 2.84, *p*<0.05) and *Per2* (*F*_3,31_ = 15.30, *p*<0.001), but not *Rev-erbα*. *Per1* varied over time in both the saline (*F*_3,31_ = 3.38, *p*<0.05) and TURP-treated groups (*F*_3,31_ = 3.72, *p*<0.01). Post hoc tests revealed a significant decrease in the TURP-treated group only at ZT16 TOS, the peak time of *Per1* gene expression (*p*<0.001). Similarly, *Per2* also varied over time in both the saline (*F*_3,31_ = 12.02, *p*<0.001) and TURP-treated groups (*F*_3,31_ = 34.12, *p*<0.001), and was suppressed in the TURP-treated group at ZT16, ZT20 and ZT0 TOS (*p*<0.01).

In the heart, there was a significant Time×Condition interaction only for *Per2* (*F*_3,31_ = 2.82, *p*<0.05). The significant variation over time in the saline controls (*F*_3,31_ = 6.68, *p*<0.001) remained after TURP treatment (*F*_3,31_ = 3.35, *p*<0.01). There was a decrease of *Per2* expression at the endogenous peak of expression 14 h after treatment (ZT16 TOS; *p*<0.05) and an increase in expression 10 h after treatment (ZT12; *p*<0.01). There was no significant interaction for either *Per1* or *Rev-erbα*. However, for *Per1* there was a trend for the main effect of Condition (*F*_1,31_ = 4.03, *p* = 0.0532), and the variation over time in the saline controls (*F*_3,31_ = 2.17, *p*<0.05) was lost after TURP-treatment (*F*_3,31_ = 0.86, *p*>0.05). In the kidney and spleen, there were no significant main effects or Time×Condition interactions for any of the clock genes.

#### In vivo experiment 2: The effect of injection time on TURP-induced changes of clock gene expression

The previous experiment showed a tissue-specific effect of TURP treatment on clock gene expression. However, treatment time may also affect the magnitude of the response. To address this, rats were treated at 4 time points over 24 h and sacrificed 10 h later, corresponding with the time of maximal febrile response [27] (Fig. 1).

**Figure 4:**
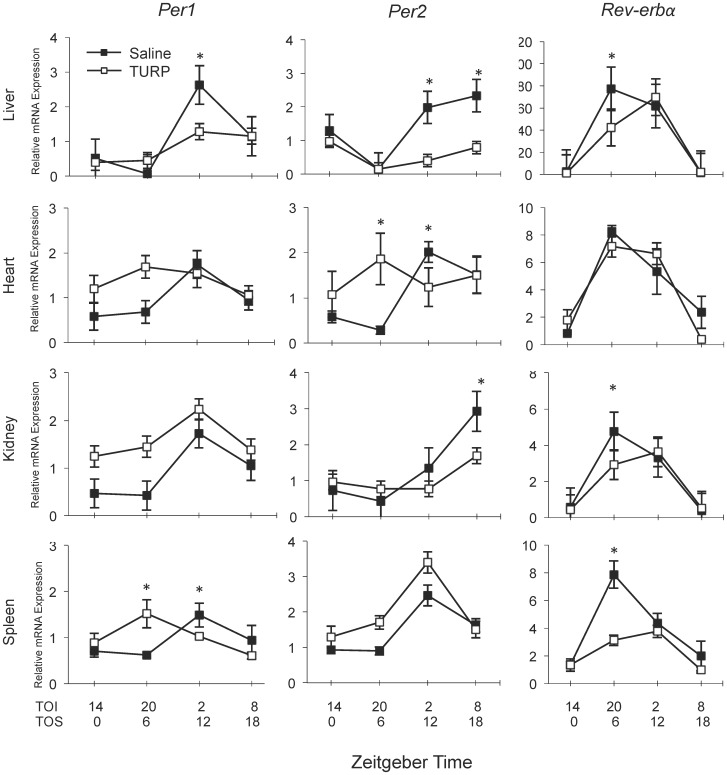
Time-dependent effect of TURP treatment on peripheral clock gene expression. Animals were treated with either TURP or saline at ZT4, ZT20, ZT2 and ZT8 (time of injection, TOI) and sacrificed 10 h later at ZT0, ZT6, ZT12 and ZT18 (time of sacrifice, TOS), respectively. Tissues were harvested upon sacrifice and RNA extracted. The relative expression of the clock genes *Per1, Per2* and *Rev-erbα* in the liver, heart, kidney and spleen is represented. Gene expression is relative to one arbitrarily chosen sample in the ZT0 TOS group. Closed boxes represent saline-treated animals and open boxes represent TURP-treated animals. Two-way ANOVA Time×Condition *p*<0.05 for *Per1* in liver and spleen, *Per2* in liver, heart and kidney, *Rev-erbα* in liver, kidney and spleen, *p*>0.05 for the others (p = 0.0862 for *Per1* in the kidney); Tukey post hoc tests between saline and TURP-treated groups at each time point (done for graphs where there was a Time×Condition interaction) *p<0.05; n = 5. Body temperature and cytokine data for this experiment can be found in Figs 2 and S2.

Considering the saline-treated controls in all tissues, *Per1* peaked around ZT12, *Per2* between ZT12 and ZT18, and *Rev-erb-alpha* at ZT6 (Fig. 4). In general, these phases of expression are consistent with previous reports [43]–[45].

In the liver, there was a significant Time×Condition interaction for *Per1* (*F*_3,31_ = 3.79, *p*<0.05) and *Per2* (*F*_3,31_ = 5.63, *p*<0.01) (Fig. 4). Post hoc analysis showed that TURP treatment significantly downregulated both *Per1* and *Per2* at ZT12 TOS (*p*<0.01). Further, *Per2* was also attenuated at ZT18 TOS (*p*<0.001). Consequently, the effect of time on *Per1* and *Per2* expression was lost in TURP-treated animals (*F*_3,31_ = 2.53, *p*>0.05; and *F*_3,31_ = 2.94, *p*>0.05, respectively). *Rev-erbα* expression also showed a significant Time×Condition interaction (*F*_3,31_ = 3.90, *p*<0.05). Although the variation over time remained significant in the TURP-treated group (*F*_3,31_ = 23.63, *p*<0.001), post hoc analysis revealed a significant decrease at ZT6 TOS (*p*<0.01).

In the heart, there was a significant Time×Condition interaction only for *Per2* (*F*_3,31_ = 5.96, *p*<0.01). Post hoc tests revealed that TURP treatment increased *Per2* expression at ZT6 TOS compared to saline controls (*p<*0.05, Fig. 4). Similarly to the liver, *Per2* expression was blunted at ZT12 TOS (*p*<0.05). Again, the significant effect of time in the saline controls of *Per2* (*F*_3,31_ = 7.93, *p*<0.001) was lost in the TURP-treated group (*F*_3,31_ = 1.42, *p*>0.05). Although there was no significant interaction for *Per1*, the significant time effect in the saline group (*F*_3,31_ = 3.26, *p*<0.05) was lost in the TURP group (*F*_3,31_ = 1.11, *p*>0.05). *Rev-erbα* showed a time effect (F_3,31_ = 8.61 p<0.001), but remained unaffected by TURP treatment.

In the kidney, there was a significant Time×Condition interaction for *Per2* (*F*_3,31_ = 5.42, *p*<0.01) and *Rev-erbα* (*F*_3,31_ = 5.91, *p*<0.01). *Per2* maintained its variation over time in both the saline (*F*_3,31_ = 21.87, *p*<0.001) and TURP groups (*F*_3,31_ = 2.99, *p*<0.05), but there was a significant reduction of *Per2* expression at ZT18 TOS (*p*<0.001). Similar to the liver, *Rev-erbα* expression was reduced at ZT6 TOS in the TURP-treated group (*p<*0.001). Although there was no significant interaction for *Per1*, there was a trend for the main effect of Condition (*F*_1,31_ = 3.14, *p* = 0.0862).

In the spleen, there was a significant Time×Condition interaction for *Per1* (*F*_3,31_ = 4.60, *p*<0.01) and *Rev-erbα* (*F*_3,31_ = 5.58, *p*<0.01), but not for *Per2*. *Per1* varied significantly over time in both the saline (*F*_3,31_ = 3.67, *p*<0.05) and TURP groups (*F*_3,31_ = 3.50, *p*<0.05), and post hoc analyses revealed an increase at ZT6 TOS (*p<*0.01) and a reduction at ZT12 TOS (*p<*0.05) in the TURP group compared to the saline group. Finally, *Rev-erbα* was reduced at ZT6 TOS (*p<*0.001), similar to the kidney and liver.

### *In vivo* experiment 3: The Effects of anti-inflammatory IL-1Ra Treatment on TURP-Induced Changes of Clock Gene Expression

IL-1Ra is an anti-inflammatory cytokine upregulated following TURP treatment and is necessary to moderate the febrile response [46]. IL-1Ra is a competitive antagonist of the IL-1 receptor [36] and consequently, it blocks IL-6 induction and fever in the TURP model by acting on IL-1 receptor at the site of localized inflammation [36]. The co-injection of hrIL-1Ra and TURP was used to assess the contribution of IL-6 to clock gene expression changes. At ZT2, animals were treated with either saline or TURP (I.M.) and in each group, the animals were also injected several times with either saline or hrIL-1Ra (I.P.). Two times of sacrifice were investigated based on the times of greatest change in Experiments 1 and 2: ZT12 and ZT16 TOS (10 h and 14 h after treatment, respectively). Altogether there were 4 groups per time point of sacrifice: saline-saline, saline-hrIL-1Ra, TURP-saline, and TURP-hrIL-1Ra.

#### IL-1Ra treatment attenuates fever induction

**Figure 5:**
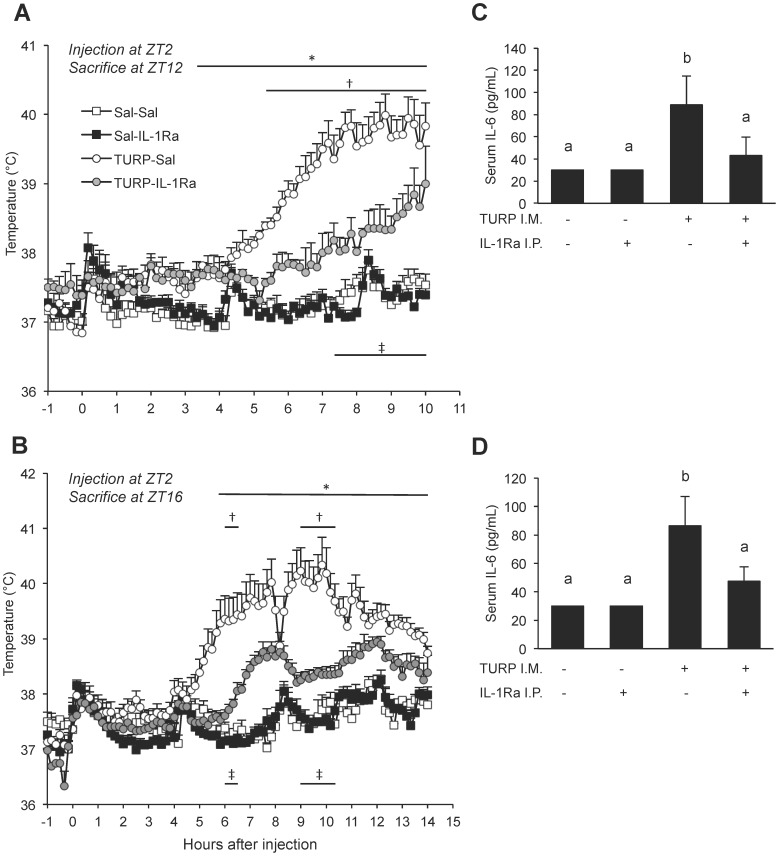
Fever and IL-6 induction after TURP treatment with or without IL-1Ra co-treatment. All animals were treated with either saline or TURP at ZT2 and sacrificed at either (A, C) ZT12 or (B, D) ZT16. Animals in each group were further treated with either saline or hrIL-1Ra 0, 4, and 8 h after the initial treatment (for animals sacrificed at ZT12) or 0, 4, 8, and 12 h after the initial treatment (for animals sacrificed at ZT16). (A, B) Core body temperature was monitored beginning 1 h before treatment and every 10 min after treatment until sacrifice. At both the ZT12 and ZT16 points of sacrifice there are 4 groups, depicted in the inset of panel A. Three-way ANOVA Time×Condition for TURP and IL-1Ra: *p*<0.001 for both the ZT12 and the ZT16 groups, simple effects of Condition or Time also *p*<0.001 in both cases, Tukey post hoc tests between conditions all *p*<0.001, except for ZT12, saline-saline vs. saline-IL-1Ra (p*<*0.05) and for ZT16, saline-saline vs. saline-IL-1Ra (p*>*0.05); n = 5. Significance in Tukey post hoc tests between the saline-saline and TURP-saline groups is represented with *, between TURP-saline and TURP-IL-1Ra with †, and between saline-IL-1Ra and TURP-IL-1Ra with ‡. (C, D) Serum IL-6 was assessed by ELISA at sacrifice (ZT12 in C, ZT16 in D). One-way ANOVA for the four groups *p*<0.01; Tukey post hoc tests between groups, different letter *p*<0.05, share one letter *p*>0.05; n = 5. Clock gene expression data for this experiment can be found in Fig. 6.

At ZT12 (Fig. 5A) there was a significant Time×Condition interaction (*F*_198,670_ = 6.891, *p*<0.001). Post hoc tests revealed the expected fever induction upon TURP treatment alone (between the saline-saline and TURP-saline groups, *p*<0.001), starting 3.33 h after treatment. Co-treatment with IL-1Ra led to an attenuated fever induction (between the TURP-saline and TURP-IL-1Ra groups, *p*<0.001), a difference that was significant from 5.33 h after treatment, lasting until sacrifice. Under TURP-IL-1Ra co-treatment, temperature was still significantly elevated compared to the control without TURP (*p*<0.001). Similarly, at ZT16 (Fig. 5B), there was a significant Time×Condition interaction (*F*_267,1237_ = 3.259, *p*<0.001). Here also, fever induction upon TURP treatment was significant (between the saline-saline and TURP-saline groups, *p*<0.001), a decrease of fever was observed upon co-treatment with IL-1Ra (between the TURP-saline and TURP-IL-1Ra groups, *p*<0.001), but to levels that were still significantly higher than controls without TURP (*p*<0.001).

#### IL-1Ra treatment blunts IL-6 induction

There was a significant effect of Condition on IL-6 levels at both TOS ZT12 (*F*_3,32_ = 9.27, *p*<0.01) and ZT16 (*F*_3,32_ = 8.63, *p*<0.01) (Fig. 5C, D). At both ZT12 and ZT16, there was a significant rise in IL-6 in the TURP-saline group compared to the saline-saline control. While there was no change in IL-6 levels in the saline-IL-1Ra group compared to the saline-saline group, IL-1Ra reduced TURP-mediated IL-6 induction at ZT12 and ZT16 (*p*<0.05) to the level of the saline-saline group (*p*>0.05) (Fig. 5C, D).

There was a significant effect of Condition on IL-1Ra levels at both TOS ZT12 (*F*_3,32_ = 8.40, *p*<0.001) and ZT16 (*F*_3,32_ = 8.04, *p*<0.001) (Fig. S3). The ELISA assay is specific to rat IL-1Ra and does not cross-react with exogenous hrIL-1Ra. Accordingly, there was no difference in the ZT12 sacrifice group between animals treated with IL-1Ra and the corresponding saline-treated controls. There was also the expected increase of IL-1Ra in the TURP-saline group compared to the saline-saline control (Fig. S3A). At ZT16, there was a significant elevation of IL-1Ra at ZT16 upon IL-1Ra administration, and an apparent but insignificant increase in IL-1Ra in the TURP-saline group compared to the saline-saline control (Fig. S3B).

#### The effects of TURP on liver clock gene expression are not reduced upon IL-1Ra treatment

**Figure 6:**
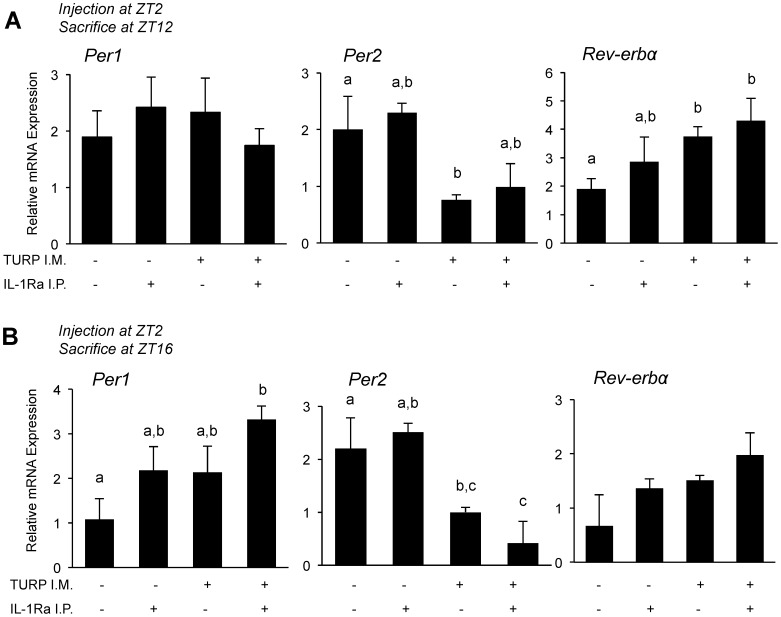
Changes in clock gene expression after TURP treatment with or without IL-1Ra co-treatment. All animals were treated with either saline or TURP at ZT2 and sacrificed at either (A) ZT12 or (B) ZT16 with IL-1Ra treatment 0, 4, 8, and 12 h after the TURP treatment (12 h IL-1Ra treatment not done in the group of rats sacrificed at ZT12). At sacrifice, the liver was harvested and relative expression of the clock gene *Per1, Per2* and *Rev-erbα* was determined. One-way ANOVA for the four groups *p*<0.05 for *Per2* and *Rev-erbα* at ZT12 and *Per1* and *Per2* at ZT16, *p*>0.05 for the others; Tukey post hoc tests between groups (when ANOVA shows significant interaction), only different letters between groups *p*<0.05, share one letter *p*>0.05; n = 5. Body temperature and cytokine data for this experiment can be found in Figs 5 and S3.

As the animals in this experiment were all treated with either saline or TURP at ZT2, we expected an effect similar to Experiments 1 and 2 of the TURP injection on clock gene expression in the liver at ZT12 and ZT16 (10 h and 14 h after TURP treatment, respectively). In general, we expected a decrease in *Per* gene expression at ZT12 and ZT16 and no change in *Rev-erbα.* Unexpectedly, there was no change among the four conditions for *Per1* at ZT12 (*F*_3,32_ = 0.41, *p*>0.05; Fig. 6A, left panel) while at ZT16, even if there was an effect of Condition (*F*_3,32_ = 3.84, *p*<0.05) there was no significant difference between the saline-saline and saline-TURP conditions (Fig. 6B, left panel).

However, as expected, *Per2* showed an effect of Condition at both ZT12 (*F*_3,32_ = 3.42, *p*<0.05; Fig. 6A, middle panel) and ZT16 (*F*_3,32_ = 5.89, *p*<0.01; Fig. 6B, middle panel), and in both cases significantly reduced mRNA levels in the TURP-saline group compared to the saline-saline group. Based on the suppression of IL-6 levels upon hrIL-1Ra treatment (Fig. 5C, D), if IL-6 serves as a mediator for the effects of TURP on clock genes, one would predict that these latter effects be blunted upon hrIL-1Ra treatment. Interestingly though, with the addition of hrIL-1Ra treatment, there was no change in *Per2* expression compared to the TURP-saline group (Fig. 6A and B, middle panels), indicating that neither systemic fever nor systemic IL-6 induction contributes to liver *Per2* expression in this model.

For *Rev-erbα,* there was an effect of Condition at ZT12 (*F*_3,32_ = 3.21, *p*<0.05), with a significant elevation in the TURP-only group compared to the saline controls, and no change upon addition of IL-1Ra at ZT12 (Fig. 6A, right panel). On the other hand, there was no effect of Condition at ZT16 (*F*_3,32_ = 0.84, *p*>0.05; Fig. 6B, right panel).

### Effect of IL-6 on a Liver-derived Cell Line *in vitro*

**Figure 7:**
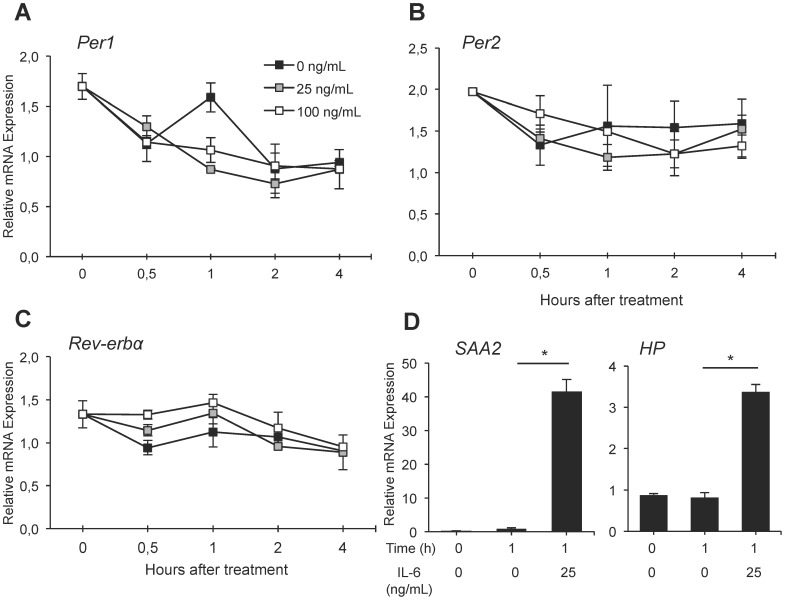
The effect of IL-6 on clock gene expression in HepG2 cells. (A, B, C) HepG2 cells were treated with 0, 25, or 100 ng/mL of IL-6 and mRNA was extracted 0.5, 1, 2, and 4 h after the start of the treatment. Relative expression of *Per1* (A), *Per2* (B) and *Rev-erbα* (C) was determined. Dose of IL-6 are depicted in the inset of panel A. Two-way ANOVA Time×Condition *p*>0.05 for all three genes. (D) Expression of *SAA2* (serum amyloid A2) and *HP* (haptoglobin) in selected samples of the same experiment as in (A, B, C). Each data point is the average of 3 culture wells. Student t test between the 1 h-0 ng/mL and 1 h-25 ng/mL groups *p<0.05; n = 5.

To assess the direct effect of IL-6 on clock gene expression in hepatocytes, human hepatoma cells (HepG2) were treated with 0, 25 and 100 ng/mL of IL-6 and clock gene expression was assessed for up to 4 h after treatment. These IL-6 concentrations were previously shown in primary hepatocyte cells to elicit the an upregulation of the acute phase gene *CINC-1* in a similar magnitude as that induced during an inflammatory challenge *in vivo* [47]. As shown in Fig. 7A–C, there was no Time×Condition interaction for the expression of *Per1* (*F*_8,19_ = 1.56; *p*>0.05), *Per2* (F_8,19_ = 0.89; *p*>0.05) or *Rev-erbα* (F_8,19_ = 0.60; *p*>0.05). This is consistent with our *in vivo* data (Figs 5 and 6) pointing to mediators others than IL-6 for the acute effect of TURP treatment on liver clock gene expression. To ensure that IL-6 was able to invoke a response in these cells under our experimental conditions, we confirmed that gene expression for the acute phase proteins serum amyloid A2 (*SAA2*) and haptoglobin (*HP*) was upregulated upon IL-6 treatment (*SAA2,* 0 vs. 25 ng/mL at 1 h, *t*_4_ = 6.70, *p*<0.01; *HP,* 0 vs. 25 ng/mL at 1 h, *t*_4_ = 7.65, *p*<0.01; Fig. 7D), as previously shown [48], [49].

## Discussion

We report that TURP-induced inflammation produces time-, gene-, and tissue-specific changes in clock gene expression in rat peripheral tissues. Interestingly, alterations in liver clock gene expression correlate with changes in IL-6 expression. However, by co-treatment with the anti-inflammatory IL-1Ra, we show that systemic IL-6 does not seem to contribute to the suppression of *Per2* in the liver. Accordingly, the *in vitro* cell culture study also suggests that IL-6 does not act directly on liver cells to induce changes in clock gene expression.

Studies show that fever parallels IL-6 induction in the TURP model [27], [50]. This is indeed what we have observed when TURP treatment was done in the morning (*in vivo* experiment 1, Fig. 1). However, by comparing the outcome of treatment at different times of day (*in vivo* experiment 2, Fig. 2), we demonstrate that the serum concentration of IL-6 ten hours after TURP treatment is dependent on the time of injection. On the other hand, fever remains largely independent of TURP injection time (except the slight reduction upon sacrifice at ZT0). The magnitude of the systemic fever response was shown by others to be independent of the time of LPS treatment [51], although the rise in temperature in the brain is dependent on this timing of treatment [52].

A possible explanation for the time-dependent induction of IL-6 could lie in the balance of rhythmic humoral factors. One possibility is the balance of melatonin and glucocorticoids, rhythmic hormones that are immune-activating [53] and immune-suppressing [54], respectively, which might be differentially regulated by the treatment at different times of day. Melatonin was also shown to act on LPS-induced intracellular signalling [55]. Another possibility for the time-dependency of IL-6 response to TURP is a circadian regulation of cytokine secretion by immune cells upon stimulation by a pro-inflammatory agent, as was shown for the LPS treatment of macrophages [56]–[58] and the activation of splenocytes through toll-like receptor 9 [59].

We observed large TURP-mediated changes in clock genes expression that were time-, tissue- and gene-dependent (Figs 3 and 4). *Per1* and *Per2* gene expression in the liver was downregulated at their normal peak (early dark phase) upon morning TURP treatment. In contrast, the effects on *Per* genes in other tissues were stronger when treatment occurred in the night, with an elevation of the expression nadir in the middle of the light phase. Effects on *Rev-erbα* mRNA levels were more subtle, but included a similar decrease of peak mRNA levels in the daytime after night-time treatment in the liver, kidney and spleen.

LPS and TURP constitute different models of inflammation: LPS provokes a fast systemic inflammatory response while TURP induces a slower response to a localized inflammation. Despite these differences, clocks respond in a similar manner to both agents. Notably, *Per1*/*Per2* RNA levels at their peak expression in the early dark phase are blunted by LPS [19], [20] and by TURP (Figs 3 and 4) in the liver. In addition, both LPS and TURP invoke tissue-specific effects on peripheral clock genes [20] (Figs 3 and 4). However, the limited number of circulating mediators in TURP-induced inflammation and its slower time-course make this model superior to tease apart the mechanisms accounting for the effects. In this respect, in the liver and to a certain extent in the heart, the maximal change in IL-6 corresponds with the reduced peak of *Per* gene expression. This suggests that IL-6 may contribute to the changes in clock gene expression induced by TURP treatment.

To test the influence of proinflammatory factors on clock gene expression in the TURP model, animals were treated with the anti-inflammatory cytokine IL-1Ra. IL-1Ra blocks the binding of IL-1β to its receptor, thus effectively eliminating the upregulation of systemic IL-6 as IL-1β is required for the upregulation of IL-6 in the TURP model [28]. Using this model, there was an expected attenuation of liver *Per2* expression in the TURP-treated groups sacrificed both 10 and 14 h after treatment (ZT12 and ZT16 TOS); however unexpectedly, *Per1* was not downregulated at either time point (Fig. 6). Experiment 1 demonstrated that the downregulation of *Per1* is transient (compared to the sustained downregulation of *Per2*), thus it is possible that our sampling missed the expected suppression of *Per1.* Considering *Per2* however, we observed that hrIL-1Ra treatment did not rescue the TURP-induced suppression despite the strong attenuation of IL-6 at sacrifice. This suggested that systemic IL-6 elevation did not induce the changes in liver clock gene expression.

Previous studies have shown that TNFα, but not IL-6, suppresses clock gene expression in fibroblasts [22]. In another study, IL-6 used at a higher concentration downregulated *Per1* promoter activity in fibroblasts [25]. We determined that systemic IL-6 does not affect *Per2* expression in the liver, but it remains possible that a tissue-specific regulation of IL-6 could contribute to clock gene regulation. In one study, it was found that *IL-6* mRNA was upregulated in the muscle tissue where TURP was injected, but downregulated in the liver [40]. Also, IL-6 in the interstitial fluid of adipose tissue is 100 times higher than in the serum and acts as a potent paracrine signaling agent [60]. Finally, it was shown that in response to LPS, IL-6 levels in spleen lymph were enriched compared to those in serum [61]. These local IL-6-enriched microenvironments could uniquely influence clock gene expression in particular tissues.

The action of IL-6 could also depend on the differential expression of its receptor. IL-6 can act via IL-6 receptor (IL-6R), which dimerizes with gp130 [62], or by binding to the soluble IL-6R (sIL-6R), which then binds to gp130 [63]. Both cases initiate the JAK-STAT pathway. While only hepatocytes and some immune cells contain IL-6R [64], gp130 is ubiquitously expressed and can thus bind sIL-6R in various tissues [63]. It is possible that any of these proteins (IL-6R, gp130 or sIL-6R) are expressed rhythmically, thereby gating the response to IL-6 to induce different magnitudes of response depending on the time of day. To test whether IL-6 can act directly on the clock in liver cells, we treated liver carcinoma cells (HepG2) with IL-6 (Fig. 7). The absence of effect on clock genes in this assay suggests that IL-6 does not have a direct effect on clock gene expression in liver cells.

Apart from IL-6, other humoral factors may impinge on the clock, as it has been shown that other cytokines can be upregulated in various tissues in the TURP model. Interferon(IFN)-γ and IFN-α treatment at ZT12 (but not ZT0) disturbs *Per* gene expression in both the liver and SCN [24]. TNFα was shown to suppress clock gene expression both in synchronized fibroblasts and in the liver [22]. Another proinflammatory factor, PGE_2_, was shown to have clock gene-modulating effects, inducing transient *Per1* expression in cultured fibroblasts and time-dependent circadian phase shifts in peripheral clocks [65], [66]. Finally, leptin is an energy-regulating hormone that can act as a proinflammatory cytokine during a febrile response [67] and rises in response to inflammatory signals [68] including TURP [69]. Interestingly, leptin treatment can alter the phase of the clock both centrally and peripherally [70], and mice lacking functional leptin (*ob/ob* mice) exhibit disrupted clock gene expression in both adipose tissue and liver [71]. Given that leptin serum levels vary over the day and are controlled by the SCN central clock [72], this hormone could possibly gate the response to inflammatory signals, thus imposing a time-dependent regulation on clock gene expression.

In addition to humoral factors, neuronal connections and systemic cues such as body temperature or pain might mediate the effects of TURP on clock genes. To our knowledge, there are no reports of an effect of pain on clock genes. Moreover, the levels of pain in our TURP inflammation model is very low compared to established models of pain, which makes it an unlikely mediator for the strong effects we see in clock gene expression. Cycles of temperature changes are thought to participate in circadian gene expression in the periphery [73]–[75], with a mechanism that involves the heat-sensing transcription factor Heat Shock Factor 1 [74]–[76] and the cold-sensing factor Cold-Inducible RNA-Binding Protein [77]. In fact, *Per2* itself has HSF1 binding sites in its promoter [76], [78]. However, while fever induction is relatively independent of the time of injection, clock gene expression changes are injection time-dependent (Figs 2 and 4). Furthermore, our data showed that *Per2* mRNA repression by TURP treatment was unchanged even when IL-1Ra co-treatment strongly reduced fever (Figs 5A, B and 6). Therefore, although we cannot exclude that the remaining fever following co-treatment with IL-1Ra might still be sufficient to affect clock genes, we believe that temperature is an unlikely mediator for the clock gene expression changes we have observed in the liver. Regarding a possible neuronal influence, sympathetic and parasympathetic pathways are likely to be involved in the entrainment of peripheral circadian clocks as there are many neuronal connections from the SCN to peripheral tissues including the liver, kidney, heart, muscle and spleen [2], [79]. For example, *Per1* gene expression is quickly induced in the adrenal glands upon light stimulation, an induction that is lost in animals with adrenal denervation [80].

It was found that the clocks in different tissues have different sensitivities to humoral cues and it is likely that balance of neuronal and humoral cues together coordinate tissue-specificity in peripheral clock entrainment [3], [5]. In particular, it was previously shown that the liver and kidney seem to be more sensitive to humoral cues, whereas the heart, spleen and muscle require other input – probably neuronal – to fully entrain their clocks [5]. Such differences in the response of tissues to different types of cues might contribute to the tissue-specific response to TURP that we have observed.

Here, we have found that TURP-mediated inflammation induces time-, tissue- and gene-specific effects in peripheral circadian clocks. This localized inflammation model will help to identify the mediators underlying the impact of inflammation on circadian rhythms. Characterizing the changes in peripheral clock gene expression upon inflammation is essential to decipher the mechanisms behind time-dependent susceptibilities to inflammatory responses.

## Supporting Information

Figure S1 Cytokine induction over time after TURP treatment at ZT2. Animals were treated with either saline or TURP at ZT2 (0 hours) and sacrificed at 6 different time points ZT4, ZT8, ZT12, ZT16, ZT20, and ZT0 (time after sacrifice, TOS) corresponding to 2, 6, 10, 14, 18, and 22 h after injection (HAI), respectively. (A) IL-1Ra and (B) TNFα levels in serum at sacrifice were measured by ELISA. The assay detection limit is 30 pg/mL. Closed boxes represent saline-treated animals and open boxes represent TURP-treated animals. Two-way ANOVA Time×Condition, *p*>0.05 for IL-1Ra, *p*<0.001 for TNFα; Tukey post hoc tests between the saline- and TURP-treated groups *p<0.05; & *p* = 0.0637; n = 4.
(TIFF)

Figure S2 Time-dependent effect of TURP injection on cytokine levels. Animals were treated with either TURP or saline at ZT14, ZT20, ZT2, or ZT8 (time of injection, TOI) and sacrificed 10 h later at ZT0, ZT6, ZT12 and ZT18 (time of sacrifice, TOS), respectively. (A) IL-1Ra and (B) TNFα levels in serum at sacrifice were measured by ELISA. Closed boxes represent saline-treated animals and open boxes represent TURP-treated animals. Two-way ANOVA Time×Condition, *p*<0.001 for IL-1Ra, *p*<0.05 for TNFα; Tukey post hoc tests between the saline- and TURP-treated groups *p<0.05; n = 5.
(TIFF)

Figure S3 IL-Ra induction after TURP treatment with or without hrIL-1Ra co-treatment. All animals were treated with either saline or TURP at ZT2 and sacrificed at either ZT12 or ZT16 time of sacrifice (TOS) with additional IL-1Ra treatment at 0, 4, and 8 h (ZT12 TOS) and 0, 4, 8 and 12 h (ZT16 TOS) after the initial treatment. One-way ANOVA for the four groups, *p*<0.001; Tukey post hoc tests between groups, only different letters between groups *p*<0.05, same letter *p*>0.05; n = 5. In all samples, TNFα was not induced above the detection limit (results not shown).
(TIFF)

Table S1 Primer sequences used in the SYBR Green quantitative PCR assays.
(PDF)

Table S2 Controls genes selected by GeNorm for each in vivo experiment.
(PDF)
