# Supplementary material for: Time-Dependent Effects of Localized Inflammation on Peripheral Clock Gene Expression in Rats
Source: PLoS One. 2013 Mar 20;8(3):e59808. doi: 10.1371/journal.pone.0059808 (PMC3603876; doi:10.1371/journal.pone.0059808)
Supplement: Table S1 — Primer sequences used in the SYBR Green quantitative PCR assays. (PDF) [file pone.0059808.s004.pdf]

S. Westfall; Effects of inflammation on clock gene expression

Table S1. Primer sequences used in the SYBR Green quantitative PCR assays.

Gene	Forward Primer	Reverse Primer
**Clock Genes**		
Per1	5' – TCACCTTCCCTGTTTTGTCC – 3'	5' – TGCTGTTTGCATCAGTGTCA – 3'
Per2	5' – CACCCTGAAAAGAAAGTGCGA – 3'	5' – CAACGCCAAGGAGCTCAAGT – 3'
Rev-erbα	5' – ACAGCTGACACCACCCAGATC – 3'	5' – CATGGGCATAGGTGAAGATTTCT – 3'
**Control Genes**		
H1	5' – GAACGCCGACTCCCAGATC – 3'	5' – CCCCTTTGGTTTGCTTGAGA – 3'
Hprt	5' – ATGGGAGGCCATCACATTGT – 3'	5' – ATGTAATCCAGCAGGTCAGCAA – 3'
Gapdh	5' – TGCCAAGTATGATGACATCAAGAAG – 3'	5' – AGCCCAGGATGCCCTTTAGT – 3'
Ubi	5' – CTCCAACAGGACCTGCTGAAC – 3'	5' – CTGAAGAGAATCCACAAGGAATTGA – 3'
Tbp	5' – TTCGTGCCAGAAATGCTGAA – 3'	5' – TGCACACCATTTTCCCAGAAC – 3'
