# Supplementary material for: Time-Dependent Effects of Localized Inflammation on Peripheral Clock Gene Expression in Rats
Source: PLoS One. 2013 Mar 20;8(3):e59808. doi: 10.1371/journal.pone.0059808 (PMC3603876; doi:10.1371/journal.pone.0059808)
Supplement: Table S2 — Controls genes selected by GeNorm for each in vivo experiment. (PDF) [file pone.0059808.s005.pdf]

Table S2. Controls genes selected by GeNorm for each in vivo experiment.

	**Liver**	**Heart**	**Kidney**	**Spleen**
**Experiment 1**	*Ubi*<br>*Tbp*	*Histone H1*<br>*Gapdh*	*Hprt*<br>*Tbp*	*Hprt*<br>*Tbp*
**Experiment 2**	*Ubi*<br>*Tbp*	*Histone H1*<br>*Gapdh*	*Hprt*<br>*Tbp*	*Hprt*<br>*Tbp*
**Experiment 3**	*Histone H1*<br>*Hprt*	n/a	n/a	n/a
